# Bacteriophage Production Models: An Overview

**DOI:** 10.3389/fmicb.2019.01187

**Published:** 2019-06-04

**Authors:** Rodrigo García, Simone Latz, Jaime Romero, Gastón Higuera, Katherine García, Roberto Bastías

**Affiliations:** ^1^Laboratorio de Microbiología, Instituto de Biología, Pontificia Universidad Católica de Valparaíso, Valparaíso, Chile; ^2^Laboratorio de Biotecnología, Instituto de Nutrición y Tecnología de los Alimentos, Universidad de Chile, Santiago, Chile; ^3^Instituto de Ciencias Biomédicas, Facultad de Ciencias de la Salud, Universidad Autónoma de Chile, Santiago, Chile

**Keywords:** bacteriophage, bacteriophage production, bacteriophage therapy, phage therapy, phage production models

## Abstract

The use of bacteriophages has been proposed as an alternative method to control pathogenic bacteria. During recent years several reports have been published about the successful use of bacteriophages in different fields such as food safety, agriculture, aquaculture, and even human health. Several companies are now commercializing bacteriophages or bacteriophage-based products for therapeutic purposes. However, this technology is still in development and there are challenges to overcome before bacteriophages can be widely used to control pathogenic bacteria. One big hurdle is the development of efficient methods for bacteriophage production. To date, several models for bacteriophage production have been reported, some of them evaluated experimentally. This mini-review offers an overview of different models and methods for bacteriophage production, contrasting their principal differences.

## Introduction

Bacteriophages have come into focus of scientific research, as they play an important role in almost every microbial community. As viral predators of bacteria, they have a substantial influence on microbial populations and dynamics in different environments. There are several reviews dealing with the role of bacteriophages in different habitats such as the seas or the human body (Clokie and Mann, 2006; Wahida et al., 2016; Łusiak-Szelachowska et al., 2017). Since their discovery more than 100 years ago, separately by Frederick Twort and then by Felix D’Herelle (Salmond and Fineran, 2015), bacteriophages have been used in eastern European countries for medical treatment of bacterial infections, whereas in the rest of the world antibiotics were the protagonists (Myelnikov, 2018). Nowadays, as infections with multi-resistant bacteria have become a worldwide threat (Zaman et al., 2017), patients from all over the world are treated at the Eliava Institute of Bacteriophages, Microbiology, and Virology, in Tbilisi, Georgia, which has perhaps the longest experience in bacteriophage therapy (Kutateladze and Adamia, 2008), and also in the Bacteriophage Therapy Unit of the Ludwik Hirszfeld Institute of Immunology and Experimental Therapy in Wrocław, Poland (Międzybrodzki et al., 2012). The application of bacteriophages could be not only a valuable solution in the medical sector, but as well as in other fields where bacteria can have a negative impact.

Some companies in the United States such as OmniLytics Inc. (Sandy, UT, United States) and Intralytix Inc. (Baltimore, MD, United States) have developed different bacteriophage products for the application as disinfectants in the food industry that can be used against *Salmonella*, *Escherichia coli* and *Listeria monocytogenes*. In Europe, a Dutch company Micreos BV (Wageningen, Netherlands) also marketed bacteriophage products against *Salmonella* and *E. coli* and a German company, Fink Tec (Hamm, Germany), targeting *E. coli* (Moye et al., 2018). Wider application of bacteriophages is expected in the food value chain including agriculture and aquaculture, where a broad spectrum of diverse plant and fish pathogens causes significant economic losses (Buttimer et al., 2017; Doss et al., 2017).

Although some bacteriophage products are already being commercialized, an effective, constant and controllable process for bacteriophage production has yet to be achieved. The production of phages in laboratories can be considered a routine process, and protocols are well defined; however, these processes are not easily scaled up. Industrial entities have the main interest to obtain reliable methods for phage production that allow scale-up of the process. However, the solution is not easy, due to the biological nature of the system and the diverse types of interactions that occur between phages and bacteria.

There have been several attempts to generate reliable methods for bacteriophage production. Some researchers have used a theoretical approach with simulation models, while others took the practical approach through experiments. This mini-review examines selected examples of both approaches, contrasting their principal differences.

## Generalities in Bacteriophage Production

The biological nature of bacteriophages forces their reproduction in the host cell. Therefore, a method for bacteriophage production requires a production process involving at least two operating units, growth of the host bacteria and bacteriophage propagation (or infection). It is important to consider basic parameters for bacterial growth and phage infection, such as the selected substrates for the bacterium and the optimal temperature, both for bacterial growth and phage infection, since these factors may influence the infectivity of phages (Tokman et al., 2016). Similarly, is important to know the biology of the phage to be produced, including the different infection parameters such as adsorption rate, burst size and latent period; however, as will be discussed later, these parameters can change depending on the infection conditions (Santos et al., 2014). More importantly, is recommended to have a deep understanding of the specific interactions that may occur between the bacterial host and the selected phage, such as the presence of a CRISPR-cas system in the bacterium, because these factors may have a strong influence on the phage infection process (Levin et al., 2013). It is also recommended to select a non-virulent bacterial strain as host. Bacteriophage production at the industrial level will require large quantities of the host bacterium, so avoiding the use of virulent drug-resistant, and especially multi-resistant pathogens should be mandatory in a phage production process (Torres-Barceló, 2018). The same applies for bacteria carrying prophages, because they could be induced during the process, altering the final outcome (Stewart and Levin, 1984).

A reliable process for large-scale bacteriophage production can be very elusive, as data obtained in a laboratory are not always useful for scaling up biological processes (Kwok, 2010). Researchers have tried to fill this gap mainly through studies on bacteriophage production based on computer simulations, some of them validated experimentally. Here, we will analyze first theoretical studies focused on phage production models and then selected studies that have been validated experimentally. All the cases agree with the assay criteria for further purification and validation of a bacteriophage-based product, and some of them are included in both sections (Santos et al., 2014; Nabergoj et al., 2018a).

## Theoretical Models for Bacteriophage Production

To describe a process of phage production through a mathematical model it is important to define the kinetic parameters to include in the model. The three basic parameters for phage production are the populations of susceptible uninfected bacteria, phage-infected bacteria, and free phages (Krysiak-Baltyn et al., 2016). Starting from this, different models have included additional variables such as resistant uninfected bacteria (Santos et al., 2014; Chaudhry et al., 2018) or multiple bacterial species (Levin et al., 1977). All these populations interact controlled by kinetic parameters associated with bacterial growth and phage infection. It is considered well known which constants are important for bacteria; however, this is still in discussion for bacteriophages. There is consensus that adsorption constant, latency period and burst size are important variables to consider; however, their relevance in the model varies between different studies. Moreover, different authors use different nomenclature to define the kinetic parameters, which is one of the main difficulties to establish comparisons between different models and to unify the general knowledge on this topic. For instance, the adsorption rate of phages (indicator of phage particles adsorbed to bacteria) is commonly referred to by the symbol “δ”; however, Beretta and Kuang (1998) used the symbol “K,” which can be also the symbol for Monod’s constant of substrate specificity “K_s_.” Other examples of different nomenclature can be found in Table 1. As in other biological process, it is expected that authors working in the field of phage-bacteria growth models agree on a specific algebraic vocabulary or include a clear explanation of terms and units in their articles and a clear nomenclature, as recently stated by Krysiak-Baltyn et al. (2018). Based on the nomenclature used by other authors (Table 1) we propose the use of Greek characters to name the different kinetic parameters in phage reproduction. Burst size can be symbolized by β, adsorption rate by δ, eclipse time by ε and phage decay rate by λ. The only exceptions would be phage concentration, which is commonly known as “P,” and latency time, known as “L.” Uniformity in this mathematical language will facilitate the understanding and data mining for future academic or industry reviewers.

**TABLE 1:** Models of bacteriophage production.

**N°**	**Model^a^**	**Nomenclature**	**System setup**	**Specific considerations of the study**	**References**
1	$\frac{d\,P}{d\,t}=k_{A}\,N\,\left[B\,\left(t-l\right)\,P\,\left(t-l\right)\right]$ $-k_{A}\,P\,B-k_{1}\,\text{P-aP}$	P = phage concentration, t = time, k_a_ = adsorption rate, N = yield of phage particles per infected cell, B = bacteria concentration, k_1_ = rate of spontaneous inactivation of phage, l = time after infection, a = flow rate constant.	Continuous process	Considers phage decay rate, considers competition with other species of bacteria (not susceptible to phage), occurrence of phage resistant strains is discussed.	Campbell,1961
2	$p_{k}=\sum_{i=1}^{I}b_{i\,k}\,e^{-\rho \,l\,i\,k}\,\gamma_{i\,k}\,n_{i}^{\prime }\,\left(t-l_{i\,k}\right)\,p_{k}^{\prime }\,\left(t-l_{i\,k}\right)$ $-\rho \,p_{k}-\sum_{i=1}^{I}\gamma_{i\,k}\,n_{i}\,p_{k}$	p_k_ = phage k concentration, n_i_ = susceptible bacteria i concentration, l = latent period, γ = adsorption constant, ρ = rate flow of the system, b = burst size, t = time, e = consumption of resources, the ( ’ ) indicates that a function is to be evaluated at a previous point in time.	Continuous process	Considers scenarios with multiple bacterial species, discusses the presence of resistant bacteria, validated experimentally.	Levin et al.,1977
3	$\frac{d\,P}{d\,t}=b\,\lambda \,\text{I-KSP}-\mu \,P$	P = free phage, t = time, b = virus replication factor (burst size), λ = death rate constant, K = effective per bacteria contact rate constant with viruses (rate of effective contact between bacteria and virus), I = virus-infected bacteria, S = susceptible bacteria, μ = virus death rate constant.	Batch operating process	Proposes the existence of a threshold virus replication factor (burst size) required for phage survival, considers phage decay rate.	Beretta and Kuang,1998
4	$\bar{P}=-P\,w-\delta \,P\,U-\delta \,P\,I+b\,e^{-w\,L}\,\delta \,P_{L}U_{L}$	P = density of free phage, w = washout rate, δ = adsorption rate, U = density of uninfected cells, I = density of infected cells, b = burst size, subscript L = value of the variable L time units in the past, superscript dot = derivative with respect to time.	Continuous operating process	Compares a one-stage process with a two-stage process from an evolutionary perspective, validated experimentally.	Bull et al.,2006
5	$\ln\,\left(\frac{P}{P_{0}}\right)=-\delta \,\left(\frac{X_{S_{0}}}{\mu }\right)\cdot \left(e^{\mu \,t}-1\right)$	P = phage concentration, t = time, P_0_ = initial phage concentration, δ = adsorption constant, X_S_ = initial concentration of susceptible uninfected bacteria, μ = bacteria multiplication rate.	Batch operating process	Considers influence of bacterial growth rate in the phage adsorption rate, considers acquired resistance, considers variations in latent period and adsorption rate, allows for substrate influence analysis, validated experimentally.	Santos et al.,2014
6	$\frac{d\,P}{d\,t}=-K_{i,\sigma \,\left(\mu \right)}\,X_{S}\,P+\sum_{m=1}^{M}b_{m}\cdot D_{T,m}\,X_{I,m,N}$ $-d_{P}\,P\,\left(t\right)$	S = substrate concentration, D_T,m_ = aging rate of infected bacteria m, P = concentration of phages, X_S_ = concentration of susceptible bacteria, b = burst size, K_i,m_ = adsorption rate constant, T = latent period, d_P_ = decay rate of phages, μ = bacterial specific growth rate as function of substrate, N = number of steps to represent latent period, M = number of populations to represent K_i,m_, T_m_ and b_m_ as functions of μ, X_I,m,n_ = concentration of infected bacterial population m at stage n, σ(μ) = function specifying which infected population X_I,m,n_ should increase in concentration.	Two stage process with self-cycling batch reactors	Semi-continuous operation with one biorreactor for bacterial growth and a second biorreactor for phage propagation, simulation data suitable to production levels, does not consider appearance of bacteriophage resistance, variation of infections parameters as function of bacterial growth rate, considers cost of operation.	Krysiak-Baltyn et al.,2018
7	$\frac{d\,V}{d\,t}=\delta \cdot \psi \,\left(R\right)\cdot N\cdot V\cdot \left(\beta -1\right)$	V = density of phages, t = time δ = adsorption rate, ψ (R) = monod function for bacteria growth for limiting resource R, N = population of susceptible bacteria, β = burst size.	Serial transfers of batch operating process	Population of susceptible bacteria can become resistant over time, population of resistant bacteria can become susceptible, validated experimentally, does not consider latent period, adsorption rate declines with the concentration of resources.	Chaudhry et al.,2018
8	$P=\frac{D_{P}^{2}}{\delta }\cdot \frac{C\cdot \left({\mathrm{be}}^{-{\mathrm{LD}}_{p}}-1\right)-\left(\frac{D_{p}}{\delta }\right)}{C\cdot \left(1-e^{-{\mathrm{LD}}_{p}}\right)+\left(\frac{D_{p}}{\delta }\right)}$	P = free phage concentration, δ = adsorption constant, L = latent period, b = burst size, C = bacterial concentration, D_p_ = dilution rate in biorreactor “P”.	Continuous process in a cellstat scheme	Production in cellstat system considering one biorreactor for bacteria growth and a second biorreactor for phage propagation, considers host bacteria physiological state, validated experimentally.	Nabergoj et al.,2018a

*Only parameters associated with change in bacteriophage population are listed; complementary information can be found in the corresponding references. ^a^Please note that different studies use different parameters in their models, which are listed in the column nomenclature.*

Beginning with Campbell (1961), many efforts have been made to describe models of bacteriophage production, describing phage population behavior under several conditions and methods. Table 1 summarizes different phage production models, given as differential or integral equations (depending on each author’s decision), mentioning specific considerations for each model.

Phage production models are generally consistent in describing phage population change over time. This may be represented as a kinetic change in phage particles or plaque forming units (PFU) per unit of time, final concentrations obtained after a batch process, or during a period of time in a continuous process. In spite of the general consensus, these models differ in several statements. Models proposed by Campbell (1961) and Beretta and Kuang (1998) are consistent in balancing phage particles with generation terms (liberation of bacteriophage particles per unit of time) and loss of free bacteriophage due to adsorption or decay rates; these models are useful due to their simplicity and the use of standard phage growth parameters like adsorption rate, burst size and latency time, and are a quick way to simulate batch production processes, but they could not fit processes like resistant bacteria populations or phage evolution over time. These models also tend to underestimate the influence of parameters such as burst size and latent period, while more recent models have shown the importance of these parameters and how they can vary depending on other factors (Santos et al., 2014; Nabergoj et al., 2018b).

An interesting model recently proposed by Santos et al. (2014) considers the influence of bacterial growth rate on the phage adsorption constant and a normal distribution equation that rules the values of latent period, taking into account the variability in these parameters. This model has proven to be very useful because it provides the opportunity to evaluate the influence of the substrate in phage production, and including the bacterial growth rate in the model offers an indirect tool to consider the physiological state of the bacteria during the process. The dependence of bacteriophage infection parameters on bacterial growth rate was later also explored by other authors (Krysiak-Baltyn et al., 2018; Nabergoj et al., 2018b); Nabergoj and colleagues found that burst size increased linearly with bacterial growth rate, while adsorption constant and latent period decreased.

Other models have explored the influence of multiple bacterial species, and the occurrence of bacterial resistance (Levin et al., 1977; Santos et al., 2014; Chaudhry et al., 2018). Although the aim of these studies was not always to develop methods for phage production, they are useful to describe potential situations than can occur during the process. These models include variables associated with bacterial resistance selection and reversion rates as a function of the bacterial population (availability of less or more susceptible bacteria over time), defining conditions in which susceptible and resistant bacteria can co-exist, such as a strong selective disadvantage in resistant bacteria (for instance lower growth rate), and/or the existence of a spatial refuge (or density refuge) where (below which) the phage is not able to infect the bacteria. Chaudhry et al. (2018) offered an interesting explanation of how phages can persist in populations dominated by resistant bacteria, suggesting that the latter could produce susceptible bacteria at frequencies that would allow phage replication. Interestingly, this phenomenon has been suggested before (Bastías et al., 2010). The generation of phage-resistant strains in phage production systems could be a cause of concern and should therefore be included in the development of new methods, to minimize this possibility. Several authors have suggested that this problem can be avoided with the phage production setup, which will be discussed in the next section.

Another interesting study is that of Krysiak-Baltyn et al. (2018), which also incorporates variable infection parameters as a function of bacterial growth rate, and estimates operational cost and productivity in a simulated two-stage process system. One of the important conclusions of this theoretical study is that the optimal substrate concentration for bacterial growth should not be necessarily the same for bacteriophage production, and according their analysis the cost per mL of phages at a concentration of 4 × 10^10^ phages/mL could be as low as $ 1.78 × 10^–2^. It would be interesting to have an experimental validation of this estimation, and to determine how it is adapted to different economies or countries.

Finally, bacteriophage evolution must be considered in a production process as well, since the phages might increase or decrease their efficiency to infect bacteria over time (Lenski and Levin, 1985). This concept could be represented as infection rates in host-range experiments, where even methods for host-range expansion can be achieved for phage therapy applications (Mapes et al., 2016). This situation has been simulated in batch cultures, showing that appearance of phage mutants is strongly dependent on the genetic flexibility of phages (rates of mutations) (Levin and Bull, 2004). The ability to predict phage evolution during production would be helpful to set up a production process minimizing the probability of altering the lytic properties of phages. The reviewed articles show that bacteriophage production models are an important approach that can help to find the best strategies, however, they need to be validated experimentally.

## Experimental Experiences in Bacteriophage Production

There are several practical studies involving phage production. Some are focused on phage production in bioreactors, while others focus on the evaluation and optimization of the process. As expected, these experiences also consider a step of bacterial growth and phage infection/propagation in flasks and bioreactors (Table 2). This data is useful to give insights on how certain models of host-bacteriophage can be used for propagation and increase levels of phage production. The most common host-phage systems used are *E. coli* strains and their phages, probably due to the amount of information regarding these bacteria-phage systems (*E. coli* phages T3, T4, and T7) and the lack of information on other bacteria-phage systems.

**TABLE 2:** Production data available on bacteriophage production cases evaluated experimentally.

**Host – Phage system**	**Phage production and specific parameters**	**References**
*Escherichia coli* ATCC 11303 – Phage T4 ATCC 11303-B4	Productivity : 7.59 × 10^14^ PFU mol CO2^–1^ Working Volume : 1 L (fermenter). Air inflow: 0.4 vvm	Sauvageau and Cooper,2010
*Escherichia coli* strain DSM 613 bacteriophage T7	Production : 1.3 × 10^10^ PFU mL^–1^ Working Volume : 3 L (fermenter)	Smrekar et al.,2011
*Salmonella enterica* serovar Enteriditis strain S1400 *Salmonella* phage PVP-SE1	Production : 1 × 10^12^ PFU mL^–1^ Working Volume : 5 L (bioreactor). Air inflow: 1 vvm	Santos et al.,2014
XL1-Blue MRF *E. coli* – M13KE phage	Production: 5 × 10^12^ PFU mL^–1^ Working Volume: 1,2 L (flask)	Warner et al.,2014
*Escherichia coli* B strain – bacteriophage T4	Production : 1.2 × 10^16^ PFU mL^–1^ Working Volume : 8 L (Fermenter)	Sochocka et al.,2015
*Staphylococcus xylosus* CTC1642 bacteriophage phiIPLA-RODI	Production : 1 × 10^9.3^ PFU mL^–1^ Working volume: 10 mL (Flask)	González-Menéndez et al.,2018
*Escherichia coli* ATCC 11303 – Phage T3 ATCC 11303-B3	Production : 10^11^ PFU mL^–1^ Working Volume : 1 L (bioreactor)	Mancuso et al.,2018
*Escherichia coli* K12 MG1655 – Phage T4 (DSM 4505)	Phage productivity: 1⋅;10^9^ PFU mL^–1^ h^–1^ Production: 2.4⋅10^13^ PFU day^–1^ Working Volume: 1 L Dilution rate : 2.0 h^–1^	Nabergoj et al.,2018a

According to one reported case, the titers obtained can be as high as 1.2 × 10^16^ PFU mL^–1^ in a batch bioreactor (5 L) (Sochocka et al., 2015). This level of production agrees with the production needed for therapeutic purposes (>1 10^10^ PFU mL^–1^), considering purification steps, the decay rate of phages and stability or shelf life (Naghizadeh et al., 2018). Other authors have also reported promising levels of production of 5 × 10^12^ PFU mL^–1^ in 1.2 L (Warner et al., 2014), and 2.4 × 10^13^ PFU day^–1^ in 1 L (Nabergoj et al., 2018a; Table 2).

It is difficult to establish comparisons about which method could be more efficient, since they use different culture procedures and different host-bacteriophage systems. Batch culture is the cheapest (not simplest) way to produce bacteriophages, but it is highly limited by the maximum volume of the equipment available, total operation times and substrate availability (higher concentrations can be inhibitory for bacterial growth). Continuous culture has higher scalability when optimizing bacterial dilution rate via inlet and outlet flux modification. Besides, regulating the dilution rate will allow direct control over the bacterial growth rate, which has a direct influence on infection parameters like burst size, adsorption constant and latent period (Mancuso et al., 2018; Nabergoj et al., 2018b). Dilution rate can also be used to increase the productivity of the system, as was shown by Nabergoj et al. (2018a) where a maximum phage productivity of 10^9^ phages mL^–1^ h^–1^ was achieved with a low dilution rate of 2 h^–1^ in a 1 L cellstat system. A continuous operating system can be permanently operative, and is therefore the most convenient way to produce a biotechnological product for a company. However, they are hard and expensive to implement, requiring constant monitoring to maintain the steady state. A totally continuous process for bacterial growth and bacteriophage production could increase the probability of bacteriophage resistance occurrence if specific countermeasures are not adopted (Middelboe et al., 2001).

Some authors have suggested the implementation of two-stage processes, one exclusively for bacteria production and a second for propagation of phages (Schwienhorst et al., 1996; Sauvageau and Cooper, 2010; Nabergoj et al., 2018a). This can be achieved with a cellstat system, where two bioreactors are connected in series with a constant flow through the system. In this case, the flow rate between the reactors and the volume in each reactor (and dilution rate and bacterial growth rate by addition) can be controlled in order to reach maximum productivity (Nabergoj et al., 2018a). Another interesting setup proposed by Sauvageau and Cooper (2010) consists of a semi-continuous system of a two stage, self-cycling process. In this case, each stage functions similarly to a batch culture, where the bacteria are first grown separately from the phage, and then introduced to the phage propagation stage when an appropriate concentration is reached, thus allowing the initiation of the infection process using a desired multiplicity of infection (Kasman et al., 2002). This setup also has the advantage of not requiring permanent monitoring to maintain the steady state of continuous systems, and has been used to obtain productivity of 7.59 × 10^14^ PFU mol CO_2_^–1^ (Sauvageau and Cooper, 2010). Both examples, the cellstat system and the two stage self-cycling process, have the great advantage that bacteria are grown in the absence of phages, therefore bacteriophage resistance is not favored during the process.

Finally, is important to note that there are some parameters that are not always reported in studies about bacteriophage production. For instance, parameters such as aeration proportion or air inflow into the bioreactor are mentioned only in two reports (Sauvageau and Cooper, 2010; Santos et al., 2014), even though this is one of the most important parameters in the production of bacteria at the industrial level. Information about other parameters such as energy transfer, different substrate utilization, bioreactor design, agitation, propellers and materials of construction in bacteriophage production is scarce or inexistent.

## Final Conclusion

The rediscovery of the potential use of phages in a broad spectrum of applications is very exciting and promising. The evidence suggests that systems for bacteriophage production that reduce the probability of bacteriophage resistance occurring should be preferred, such as a cellstat or a two-stage self-cycling process. These options would also allow controlling variables to increase the productivity of the process. Nevertheless, models for production of bacteriophages are far from being established and can be improved in several ways. There are still many challenges to overcome. Further studies on optimized large-scale bacteriophage production, infrastructure and equipment costs, different safety concerns and application dosage are needed, and experience suggests that these challenges should be faced with collaborative efforts of academic and industrial partners.

Finally, it is important to note that most of the models for bacteriophage production can be applied within a specific range of values for parameters of phage infection and bacterial growth. Therefore, regardless of the important advances in phage production models and setups, the deep knowledge of the specific phage-bacteria system will be always the first requirement in order to establish an efficient phage production system.

## Author Contributions

RG, SL, and RB conceived the work and wrote the manuscript. KG, GH, and JR wrote the sections of the manuscript. All authors contributed to the bibliographic revision, manuscript revision, read, and approved the submitted version.

## Conflict of Interest Statement

The authors declare that the research was conducted in the absence of any commercial or financial relationships that could be construed as a potential conflict of interest.
